# Incomplete staging surgery as a major predictor of relapse of borderline ovarian tumor

**DOI:** 10.1186/1477-7819-11-13

**Published:** 2013-01-23

**Authors:** Margarita Romeo, Francesc Pons, Pilar Barretina, Joaquim Radua

**Affiliations:** 1 Medical Oncology Department, Institut Català d’Oncologia-Badalona, Carretera de Canyet s/n 08916, Badalona, Barcelona, Spain; 2 Medical Oncology Department, Institut Català d’Oncologia-L’Hospitalet, Gran Via de l’Hospitalet, 199–203, l’Hospitalet de Llobregat, Barcelona, 08908, Spain; 3 Medical Oncology Department, Hospital del Mar-Parc de Salut Mar, Passeig Marítim 25-29, Barcelona, 08003, Spain; 4 Medical Oncology Department, Institut Català d’Oncologia-Girona, Av. França s/n. 17007, Girona, Spain; 5 King’s College London, 16 De Crespigny Park, London, SE5 8AF, United Kingdom; 6 FIDMAG Research Unit, CIBERSAM, C/ Dr. Antoni Pujadas, 38, Sant Boi de Llobregat, Barcelona, 08830, Spain

**Keywords:** Borderline ovarian tumor, Relapse, Prognosis, Surgery, Staging, Recurrence, Fertility-sparing surgery

## Abstract

**Background:**

Borderline ovarian tumors (BOTs) are a subset of epithelial ovarian tumors with low malignant potential but significant risk of relapse (10% to 30%). Unfortunately, surgical prognostic factors for BOT relapse have not been clearly identified, probably due to the use of heterogeneous surgical definitions and limited follow-up. The aim of this study was to assess potential relapse risk factors using standard surgical definitions and long follow-up.

**Methods:**

All patients diagnosed with BOT for a period of more than 10 years in a single institution were included in the analysis. Complete surgical staging was defined as the set of procedures that follow standard guidelines for staging surgery (except lymphadenectomy), performed either with one or two interventions. Fertility-sparing surgeries that preserved one ovary and the uterus but included all the remaining procedures were classified as complete staging. The relationship between potential risk factors and time to BOT relapse was assessed by log-rank tests corrected for multiple comparisons and Cox regression.

**Results:**

Forty-six patients with a median follow-up of 5.4 years were included, of whom 91.3% had been diagnosed as FIGO stage I disease and 45.7% had received complete staging surgery. Five relapses were detected (10.9%), all of them in women who had been diagnosed with stage I disease and had received incomplete staging surgery. Log-rank tests confirmed the association between incomplete staging surgery and shorter time to BOT relapse.

**Conclusions:**

Complete staging surgery should be considered a cornerstone of BOT treatment in order to minimize the risk of relapse.

## Background

Borderline ovarian tumors (BOTs), which account for 15% of epithelial ovarian tumors, are defined by the presence of cellular proliferation and nuclear atypia without an infiltrative pattern or stromal invasion. Histologically, they are divided into serous (50%), mucinous (45%) and other subtypes (namely endometrioid, clear cell and Brenner). The staging system of the International Federation of Gynecology and Obstetrics (FIGO) is the same for BOT and for invasive ovarian tumors (IOTs). Remarkably, BOT’s extra-ovarian implants can be either noninvasive or invasive [1,2].

Compared to IOTs, BOTs affect younger women and are diagnosed at earlier stages, and patients have significantly better survival rates. Approximately 85% of BOTs are diagnosed at stage I, which confers a 98% overall survival rate at 5 years [3]. Beyond stage II, the 5-year overall survival is still excellent, around 85% to 92% [2,3]. However, up to 10% to 30% of patients will suffer a relapse with locoregional (pelvic or abdominal) disease, and late relapses are relatively common (>25% relapses occur after 5 years) [4-6]. Although most of these recurrences show borderline histology, up to 30% of them may be IOTs, worsening the overall survival of BOTs [7,8].

The standard of care for BOTs is complete comprehensive staging surgery, defined as the exploration of the entire abdominal cavity, bilateral oophorectomy, hysterectomy, infracolic omentectomy, peritoneal biopsies, peritoneal washings, removal of all macroscopic suspicious peritoneal lesions and, for mucinous tumors, also appendectomy. Unfortunately, only 50% of patients undergo complete staging. Pelvic and para-aortic lymphadenectomy do not increase overall survival. Similarly, neither chemotherapy nor radiotherapy are currently accepted as part of the standard care, since randomized trials have failed to show a benefit to survival [2]; chemotherapy is only considered in cases of peritoneal invasive implants [9].

The low incidence of BOTs has made it difficult to establish solid conclusions about their prognosis. Numerous studies confirm the association of several pathologic and clinical characteristics with an increased risk of BOT relapse, namely older age at diagnosis [2], invasive peritoneal implants (present in 15% to 40% of BOTs) [10], advanced stage [2,5,8], cyst rupture [11], stromal microinvasion [5,12-14] and elevated baseline CA125 [8,15-18]. Invasive peritoneal implants and an advanced stage have also been reported to decrease overall survival [2,5,8,10,18,19]. However, the prognostic impact of surgical procedures, such as fertility-sparing surgery or the surgical approach (that is, laparotomy vs laparoscopy), is currently inconclusive.

Fertility-sparing surgery (or ‘conservative’ surgery) showed an increased risk of relapse in some studies [2,19,20]. The general objective of this approach is the preservation of the uterus and the contralateral, non-affected ovary (or at least part of it) [2], but the specific definition of the procedure (that is, including omentectomy or not) depends on the surgical team. Moreover, secondary complete surgery after completing childbearing is a standard procedure in some institutions but not in others. However, overall survival seems unaffected, probably because a significant number of these relapses exclusively involve the remaining ovary and can be surgically rescued [19,20].

The findings of previous studies might also be influenced by the length of their follow-up. A follow-up period above 5 years has been claimed to be crucial in order to properly evaluate the relapse rate, due to the high number of late relapses [1,6]. Indeed, relapse rates range from 5% in series with median follow-up <3 years [21] to 30% in series with median follow-up >5 years [5-7].

The aim of this study was to assess the potential associations between the different surgical procedures and the relapse rate of BOTs. With this aim, all patients diagnosed with BOTs during an 11-year timeframe in a single institution were classified according to strict surgical definitions. Given the results of previous studies, we hypothesized an association between incomplete surgical staging and increased risk of relapse.

## Methods

All patients diagnosed with BOT from 1 January 1992 to 31 December 2002 in a single institution were included in this study. The clinical data from each participant was collected from medical files. The variables included in the analysis were: age, baseline CA125 and CA199 values, type of surgical staging (complete or not), fertility-sparing intention, surgical approach (laparoscopy or laparotomy), performance of lymphadenectomy, histology subtype (serous or mucinous), FIGO staging, intraoperative and pathologic cyst rupture, presence and type of peritoneal implants and peritoneal cytology results. Relapse and survival information was collected after at least 5 years of follow-up (31 December 2008).

Complete surgical staging was defined as the set of procedures that follow standard guidelines for staging surgery [9], independently of whether they were performed in one or two interventions (that is, a second procedure after completing childbearing). Fertility-sparing surgery was defined as a procedure in which the uterus and at least part of one ovary were preserved with the aim of preserving fertility. Fertility-sparing surgery that included all the procedures of complete surgical staging except for hysterectomy and unilateral oophorectomy, were considered as complete staging. Histological typing was performed according to the World Health Organization’s system. The staging system was based on the 2006 FIGO stage system for ovarian cancer [1,2].

Baseline CA125 and CA199 values, type of surgery, surgical approach, performance of lymphadenectomy, histology subtype, FIGO staging, intraoperative and pathologic cyst rupture, presence of peritoneal implants and their invasiveness and peritoneal washing cytology results were included as potential risk factors for relapse. The time to relapse was defined as the time from surgery to relapse.

Due to the presence of censorship (for example, because of follow-up failure or deaths from intercurrent disease), the influence of the potential risk factors on the time to relapse was assessed using survival-analysis log-rank tests, rather than simply comparing the relapse frequencies. The false discovery rate (FDR) was used to correct for multiple comparisons. A multivariate analysis of the risk of relapse, as a function of the factors found to be statistically significant prognostic factors in the univariate analysis, was performed using Cox regression. The data were analyzed using the SPSS (SPSS Inc, Chicago, IL) version 17.0 statistical package.

## Results

### Description of the sample

Forty-six women were diagnosed with BOT between January 1992 and December 2002. The median time of follow-up (from diagnosis to relapse or last date of follow-up) was 5.4 years (interquartile range: 2.5 to 6 years). Their demographic characteristics are shown in Table 1.

**Table 1:** Characteristics of the whole cohort

	**Number of women**	**%**
Median time of follow-up (years)	5.4	
Interquartile range (years)	2.5 to 6	
Mean age at diagnosis (years ± SD)	47.77 ± 16.45	
Histology (*n* = 46):		
Serous	21	45.7
Mucinous (intestinal + Müllerian)	24	52.2
Clear-cell tumor	1	2.1
CA125 or CA199 before surgery (*n* = 34):		
Elevated (>35 UNL)	27	79.4
Normal	7	20.6
Approach (*n* = 46):		
Laparotomy	40	87.0
Laparoscopy	6	13.0
Surgery (*n* = 46):		
Complete	21	45.7
Incomplete	25	54.3
Lymphadenectomy (*n* = 46):		
Pelvic +/− para-aortic	21	45.7
None	25	54.3
Intraoperative cyst rupture (*n* = 44):		
Yes	2	4.5
No	42	95.5
Pathologic cyst rupture (*n* = 46):		
Yes	10	21.7
No	36	78.3
Peritoneal implants (*n* = 46):		
Invasive	0	0.0
Non-invasive	2	4.3
None	33	71.7
Not described by surgeon	11	23.9
Peritoneal washings (*n* = 46):		
Positive	0	0.0
Negative	46	100.0
FIGO staging (*n* = 46):		
IA	23	50.0
IB	7	15.2
IC	12	26.1
≥II	3	6.5
Information not available	1	2.2

Twenty-five patients (54.3%) received incomplete staging surgery. The main reason that these surgeries were considered ‘incomplete’ was the understaging of the abdomen, including the lack of infracolic omentectomy, the incomplete exploration of the entire abdominal cavity, and the paucity of peritoneal biopsies and peritoneal washings. There was no residual macroscopic disease in any case. Lymphadenectomies were performed in 21 of the 46 patients (45.7%), all of which included both pelvic and retroperitoneal areas; the mean number of resected nodes was 12.

Most of the patients (91.3%) were diagnosed as FIGO stage I. Peritoneal implants were found in 3 patients (6.7%), none of them invasive. No affected nodes were found by the pathologic examinations of any patient. Adjuvant treatment was not administered to any patient, according to National Comprehensive Cancer Network guidelines [9].

Laparotomy was the most frequent surgical approach (87%). Intraoperative cyst rupture was observed in only 2 patients (4.5%), but the relative frequency seemed higher in those women who had received laparoscopy (1 out of 6 patients, 16.7%), though this difference did not reach statistical significance (Fisher’s exact test *P* > 0.05). No port site metastases were developed in patients operated on by laparoscopy.

Three deaths were reported: two in patients free of recurrence, and one caused by invasive tumor progression 6.7 years after the initial diagnosis (see Table 2).

**Table 2:** Description of the five recurrences

	**Age at diagnosis**	**FIGO staging**	**Intraoperative/pathologic cyst rupture**	**Approach**	**Kind of operation (none of them had any re-resection)**	**Serum marker at diagnosis**	**Years from first surgery to recurrence**	**Nature of relapses**	**Recurrence site**	**Treatment for relapse**	**Last control status**	**Time of follow-up (years)**
1	34.53	IA	No	LSC	Adnexectomy	CA199 = 7939	1.4	IOT mucinous intestinal	contralateral ovary, IA	debulking	Free of disease	6.06
		Neg washings										
						CA125 = 4387						
2	35.63	IC	No / Yes	LT	Adnexectomy		2	BOT serous	contralateral ovary	USO + TAH	Free of disease	10.14
		Neg washings										
3	20.30	IC	No / Yes	LSC	Cystectomy		2	BOT mucinous intestinal	same ovary	debulking	Free of disease	5.80
		Neg washings										
4	62.62	IC	Yes	LSC	Adnexectomy	CA125 = 554	6.2	IOT serous	pelvic lymph node IIIC	debulking + chemotherapy	Alive with disease	9.66
		Neg washings										
5	38.42	IA	No	LT	Adnexectomy		4.7	IOT mucinous intestinal	positive ascites, IIC	debulking	Dead of disease	6.69
		Neg washings										

BOT, borderline ovarian tumor; IOT, invasive ovarian tumor; LSC, laparoscopy; LT, laparotomy; USO + TAH, unilateral oophorectomy plus total abdominal hysterectomy.

The intention of surgical intervention preserving at least one ovary and the uterus was not recorded in all of the medical files of patients who underwent this type of surgery. Thus, we were unable to establish whether fertility-sparing interventions in women aged 40 to 50 who already had children should be considered as fertility-sparing surgery or whether, conversely, they were merely incomplete.

### Recurrence characteristics

Five recurrences (10.9%) were detected (Table 2). All patients who eventually relapsed had been considered stage I BOT, and none of these patients had received complete surgical staging (their interventions had been cystectomies or unilateral adnexectomies). The mean time for recurrence was 3.3 ± 2.1 years. Laparoscopy had been used in 3 cases (60%). Three relapses were invasive (stage IA mucinous IOT in the contralateral ovary, stage IIC mucinous IOT and stage IIIC serous IOT), whilst the other two had borderline histology (one mucinous in the remaining ovarian tissue and the other serous in the contralateral adnex). Thus, 3 relapses (60%) involved the remaining adnexes exclusively, and 2 (40%) were stage II or above. Salvage treatment at relapse was based on debulking surgery in all patients but one, in whom a unilateral salpingo-oophorectomy plus total abdominal hysterectomy was performed.

At the date of last control, the three women with relapse confined to adnexes were free of disease, the woman with invasive stage IIIC relapse was alive with disease and the woman with invasive stage IIC relapse had died because of disease.

### Relapse prognostic factors

**Figure 1:**
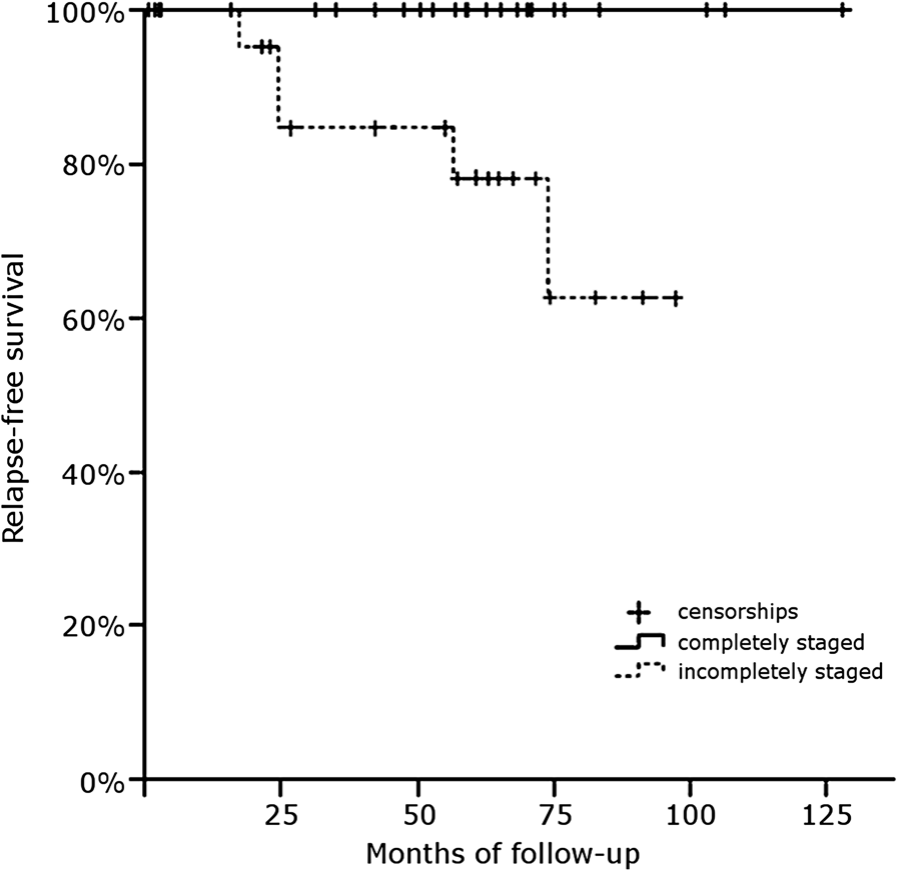
Time-to-relapse analysis in patients with complete and with incomplete staging surgery.

The time to relapse was found to be significantly lower in those patients who had undergone incomplete staging surgery (*χ*^2^ = 5. 507, degrees of freedom –df- = 1, uncorrected *P* = 0.019, FDR-corrected *P* = 0.038), in those patients operated on by laparoscopy (*χ*^2^ = 8.072, df = 1, uncorrected *P* = 0.004, FDR-corrected *P* = 0.024), and in those patients without lymphadenectomy (*χ*^2^ = 5.862, df = 1, uncorrected *P* = 0.015, FDR-corrected *P* = 0.038). The omnibus test of a Cox regression using these surgical factors was statistically significant (*χ*^2^ = 10.281, df = 3, *P* = 0.016), thus rejecting the joint hypothesis that the coefficients are all zero. Individual tests for the regression coefficients were not statistically significant (Wald = 0.003 to 0.612, df = 1, *P* > 0.05), though this could potentially be due to collinearity as these three surgical factors were not independent (*χ*^2^ = 5.217 to 15.146, df = 1, *P* = 0.001 to 0.030) (Figure 1).

No significant differences were found depending on the histology type, FIGO or the presence of pathologic cyst rupture. The presence of intraoperative cyst ruptures, positive peritoneal washing or peritoneal implants could not be included in the analysis due to the low frequencies of these events (2, 0 and 3 respectively). CA125 and CA199 values were not included because the values were missing in most of the patients who suffered a relapse.

There was no difference in survival between patients who had received complete staging surgery and patients who had not (mean survival: 9.6 vs 9.5 years, *χ*^2^ = 0.324, df = 1, *P* = 0.569).

## Discussion

Despite their excellent prognosis, BOTs are not a minor health problem as 10% to 30% of patients relapse and one third of relapses can be in form of IOTs, thus impairing overall survival [7,8]. Moreover, BOTs affect younger women than IOTs and often require surgery entailing infertility. Unfortunately, surgical prognostic factors for long-term relapse outcomes have not been clearly established, probably due to the use of different surgical definitions or to the short follow-up periods of some of the reported series.

This article presents an analysis of prognostic factors for BOT relapse in a series with a median follow-up >5 years and using the standard definition of complete comprehensive surgical staging, even in those cases where fertility-sparing surgery had been performed. The proportion of patients who suffered a relapse (10.9%) and the mean time from surgery to recurrence (3.3 ± 2.08 years) in the series presented here are similar to those reported in other long-term follow-up series [5,6,8,10,21-23]. The main finding was that incomplete staging surgery significantly increased the risk of relapse. The relapse risk was also found to be higher in patients operated on by laparoscopy (compared to laparotomy), and in patients who had not undergone lymphadenectomy.

Complete surgical staging has been reported to be an important local relapse predictive factor for nearly all tumors, and complete staging including the removal of all macroscopic implants has been demonstrated to improve overall survival in patients with IOTs [24,25]. However, the benefits of complete staging in patients with BOTs could be potentially limited because: a) it might not modify the decision regarding adjuvant treatment as this is not the standard care; and b) most recurrences can be salvaged. However, incomplete staging may prevent the possibility of detecting more aggressive disease, which could be surgically removed or be a reason to deliver chemotherapy. In a recent series of 233 patients with BOTs without re-staging procedures, those patients who had undergone complete staging suffered fewer relapses than those with incomplete staging (5.1% vs 12.3%), although this difference was not statistically significant in the multivariate analysis [8]. National Comprehensive Cancer Network guidelines [9] and other authors [2] have recommended the same complete staging procedure as is performed on patients with IOTs. Remarkably, all patients who suffered a relapse in our series had previously undergone incomplete surgical staging.

The FIGO staging classification for ovarian neoplasms is based on surgical findings and it can only be determined with complete surgical staging. In our sample, the percentage of patients with stage I disease or peritoneal implants (91.3% and 4.3% respectively) and those receiving complete surgical staging (45.7%) were in agreement with figures reported in the literature [2,5,8,22,23]. Taking into account the number of stage I cases among patients in this series with incomplete surgery, a significant number of BOTs that had been diagnosed as stage I should be better defined as *presumed* stage I. This study suggests that a higher number of complete staging procedures might result in detecting more cases with peritoneal implants and thus, that the incidence of stage I tumors in the literature might be slightly overestimated. The fact that a significant number of relapses appear in the peritoneum reinforce this idea [3,5,8,23]. The five relapses in our sample occurred for *presumed* stage I patients: the peritoneal ones were invasive while two of the three patients who had relapses in the remaining adnexes had borderline histology. The three patients with adnexes-confined relapse were free of disease in the follow-up, while the two patients who suffered an extra-ovarian relapse were not. This is concordant with the study of Wu *et al.*, in which nearly all invasive relapses occurred in the peritoneum, while most relapses with BOT histology occurred in the adnexes [8]. We hypothesize that complete surgical staging could minimize peritoneal and invasive relapse.

In our series, relapse risk was also found to be significantly higher in patients who had not undergone lymphadenectomy. It must be noted, however, that this association may be confounded by the fact that lymphadenectomies were usually performed in the context of complete staging and laparotomy, an association that might reflect the everyday work at gynecologic services until the late 1990s, when the number of systematic lymphadenectomies began to decline for patients with BOTs [26]. Indeed, lymphadenectomy is no longer recommended for BOT treatment because lymph node positivity has not been proved to affect overall survival when adjusted for FIGO staging, as reported in published retrospective series or analyses of large databases [18,27-29].

Laparotomy was also associated with decreased risk of relapse. However, this association could again be related to the accrual period (1992 to 2002), as laparotomy was the selected approach in 87% of cases. Nowadays, surgeons have improved their laparoscopy skills and a complete staging procedure with lower morbidity is feasible with this approach, although the frequency of understaging and cyst ruptures still remains high with this technique [2,22,23]. In our series, intraoperative cyst ruptures seemed more frequent in women operated on by laparoscopy (16.7%) than in those operated on by laparotomy (4.5%), like the percentages reported in 2005 by Fauvet et al. [22]. Of interest, this group found the same relapse rate between patients who had undergone laparotomy or laparoscopy [22].

Some limitations of this study must be highlighted. First, its sample size was relatively small, a condition which may have reduced the power to detect relationships between relapse incidence and factors other than surgery, such as FIGO staging. However, the small sample size also implies that the relationships detected between relapse incidence and surgical factors should probably be very strong. Second, the low numbers in some subgroups precluded the exploration of some of the potential prognostic factors such as baseline serum markers at diagnosis, intraoperative cyst rupture, peritoneal washings and the presence of implants. Fortunately, the FIGO stage may indirectly include the effects of some of these factors. Third, the three surgical factors analyzed (type of surgery, surgical approach and performance of lymphadenectomy) were not independent (*χ*^2^ = 5.217 to 15.146, df = 1, *P* = 0.001 to 0.030), so it cannot be ruled out which one or ones might be truly related to relapses and which might not. However, incompleteness of surgery may probably be the main relapse risk factor, since: a) lymphadenectomy is not considered to be a relevant prognostic factor for BOT; b) the relationship between laparotomy and completeness of staging procedures could be explained by the insufficient laparoscopic skills during the 1992 to 2002 period; and c) all patients suffering relapses in our series were incompletely staged. Finally, as mentioned earlier, this study could not separately assess the influence of fertility-sparing surgery in the prognosis of BOT.

## Conclusions

In summary, this study, though small, stresses the importance of complete staging of BOTs in order to prevent peritoneal relapse, some of which may be invasive. Moreover, complete staging enables clinicians to discover the presence of invasive implants, which may be an indication of the need for adjuvant chemotherapy. Based on the findings of this study, we suggest performing complete comprehensive staging surgery in patients diagnosed with BOT. In young patients who desire to preserve their fertility, as well as sparing the uterus and one ovary, we would suggest carefully exploring the abdominal cavity and considering peritoneal abdominal staging surgery.

Further investigations are encouraged in order to better clarify the specific roles of the different predictive factors for relapse in long-term follow-ups (>5 years).

## Abbreviations

BOT: Borderline ovarian tumor; FDR: False discovery rate; FIGO: International Federation of Gynecology and Obstetrics; IOT: Invasive ovarian tumor.

## Competing interests

The authors have no competing interests to declare.

## Authors’ contributions

The authors’ contributions were as follows: MR and JR designed the study; MR and FP retrieved the data; MR and JR conducted the statistical analyses. All of the authors were involved in drafting the manuscript or critically revising it and gave final approval of the version to be published. All authors read and approved the final manuscript.
