# Erratum to: Incomplete staging surgery as a major predictor of relapse of borderline ovarian tumor

**DOI:** 10.1186/s12957-016-0976-4

**Published:** 2016-08-25

**Authors:** Margarita Romeo, Francesc Pons, Pilar Barretina, Joaquim Radua

**Affiliations:** 1 Medical Oncology Department, Institut Català d’Oncologia-Badalona, Carretera de Canyet s/n 08916, Badalona, Barcelona Spain; 2 Medical Oncology Department, Institut Català d’Oncologia-L’Hospitalet, Gran Via de l’Hospitalet, 199-203, l’Hospitalet de Llobregat, Barcelona 08908 Spain; 3 Medical Oncology Department, Hospital del Mar-Parc de Salut Mar, Passeig Marítim 25-29, Barcelona, 08003 Spain; 4 Medical Oncology Department, Institut Català d’Oncologia-Girona, Av. França s/n. 17007, Girona, Spain; 5 King’s College London, 16 De Crespigny Park, London, SE5 8AF United Kingdom; 6 FIDMAG Research Unit, CIBERSAM, C/Dr. Antoni Pujadas, 38, Sant Boi de Llobregat, Barcelona 08830 Spain; 7 Departament de Medicina, Universitat Autónoma de Barcelona, Plaza Cívica, s/n, 08193, Bellaterra, Barcelona Spain

## Erratum

Following publication of the original article [1] it was brought to our attention that an affiliation was missing from the article. Please note that Margarita Romeo should have three affiliations: 1, 2 and 7. The additional affiliation which was missing from the original article is as follows:

^7^Departament de Medicina, Universitat Autónoma de Barcelona, Plaza Cívica, s/n, 08193 Bellaterra, Barcelona, Spain.
